# Hydrological Characteristics and Trophic Status as Dominant Drivers of Rotifer Community Composition in Artificially Created Riverine Wetlands

**DOI:** 10.3390/ani12040461

**Published:** 2022-02-13

**Authors:** Seong-Ki Kim, Jong-Hak Yun, Gea-Jae Joo, Jong-Yun Choi

**Affiliations:** 1 National Institute of Ecology, Seo-Cheon Gun 325-813, Korea; skkim@nie.re.kr (S.-K.K.); jhyun225@nie.re.kr (J.-H.Y.); 2 Department of Biological Science, Pusan National University, Busan 46241, Korea

**Keywords:** biodiversity, microhabitat, trophic state, eutrophication, macrophyte

## Abstract

**Simple Summary:**

Increased public awareness of wetlands and the importance of their conservation stipulated various preservation or improvement projects. In 2012, riverine wetlands were created in the Nakdong River in Korea to replace those damaged or destroyed by the River Refurbishment Project, but they could not be maintained as functional wetlands, owing to the long-term neglect and lack of management. A previous survey detected various problems in these wetlands, including insufficient in/outflow function, shore instability, and difficulty in introducing appropriate water sources, which cause nutrient accumulation and algal blooms. The in/outflow functions of the mainstream and tributaries were hampered by soil and plant deposition. These chemical changes influenced the community composition of rotifers. In particular, three rotifer species—*Brachionus*, *Keratella*, and *Trichocerca*—were mainly distributed in wetlands with relatively high nutrient concentrations (total nitrogen and phosphorus). Therefore, the rotifer community can be used as an indicator of the nutritional status of a wetland, and the functional state of the wetland can be understood through continuous monitoring of rotifers.

**Abstract:**

Hydrological characteristics of freshwater ecosystems are powerful determinants of the distribution of biological communities and changes in environmental factors. This study identified relationships between the wetland environment, rotifer community, and hydrological factors for 48 wetlands, to determine their impact on wetland conservation and management. Different hydrological factors produced different wetland environments, which influenced the rotifer community composition. The wetlands with “poor” “in/outflow function” and “shore stability” levels showed high conductivity, turbidity, depth, and concentrations of total nitrogen, total phosphorus, and chlorophyll *a*. In contrast, the dissolved oxygen levels and velocity were the highest in wetlands with “good” in/outflow function and shore stability variables. The nutritional status of each wetland affected the composition of the rotifer community. Some genera (*Keratella*, *Brachionus*, *Anuraeopsis*, *Trichocerca*, and *Philodina*) were found in wetlands with high concentrations of total nitrogen, total phosphorus, and chlorophyll *a*, and high turbidity and depth. In contrast*, Ascomorpha* and *Ploesoma* were found in wetlands with high dissolved oxygen levels and flow velocity. High densities of *Lepadella*, *Lecane,* and *Testudinella* were found in wetlands completely covered by macrophytes. The rotifer community distribution can be used to understand the trophic, current functional, and environmental status of wetlands.

## 1. Introduction

Wetlands are important habitats for various biological communities because of their high productivity and the characteristics of their various microhabitats [1,2]. Empirical studies have suggested that although wetlands cover only a small portion of Earth’s surface per unit area (~4–6%), they have one of the highest primary production of all ecosystems in the world (~1300 gC/m^2^/yr) [3,4]. The high biodiversity of wetlands is partly caused by the stable environment created by low flow rates. However, owing to the complex habitat structures of wetlands, controlling various biological community interactions contributes greatly to maintaining biodiversity [5]. The complex habitat structures created by aquatic macrophytes and shrubs in wetlands significantly reduce negative interactions, such as competition and predation, and allow various competitors and predators to coexist in wetlands [6]. Wetlands also contribute to ecosystem circulation and recovery through various ecological functions, such as flood control, soil stability, pollution control, material circulation, and hydrological stability [7,8]. Therefore, wetland maintenance and preservation affect the conservation and interconnectivity of various ecosystems and further contribute to regional biodiversity.

Despite their importance, the loss and destruction of wetlands in Korea continue. Wetland destruction is closely related to human activity and often results from increasing urban areas or cultivated land. In the past, there was a strong perception that wetlands were “useless land,” encouraging their reclamation and use for other purposes. Wetland loss through agricultural conversion was dominant in the early 1900s. Furthermore, reclamation caused by industrialization (construction of industrial complexes and roads) has had a significant impact since the late 1980s [9]. In 2012, through the River Refurbishment Project, large-scale maintenance and dredging were carried out in the Geum, Yeongsan, Han, and Nakdong rivers, and many wetlands located along these rivers were damaged or destroyed. Since the recognition of the importance of wetlands as biological habitats, various efforts have been made to restore them. To restore the wetlands damaged by the river maintenance project in 2012, 147 wetlands have been created along 4 major rivers in Korea. Similar restoration attempts were also reported in other countries. In the United States, a restoration project was conducted to maintain the operation of surrounding oil fields while expanding the Bolsa Chica wetland basin and restoring its tides, which had been damaged by the discovery of oil fields [10]. In Japan, restoration projects such as the Natural Restoration Promotion Act were carried out after the Kushiro Wetland suffered vegetation simplification and area reduction due to increased sediment in the wetland [11]. In Korea, the Sunpo Wetland, damaged by land cultivation, was restored with the endangered species *Brasenia schreberi* as the flagship species [12].

In wetland restoration, various environmental conditions, including hydrological factors, contribute to the maintenance and the determination of wetland type [4], which further affects the distribution of various organisms [13]. Choi et al. [14] suggested that factors such as inflow and outflow and continuous erosion of the revetment slope contribute to the changes in the chemical (e.g., dissolved oxygen (DO) and pH) and physical (e.g., habitat structure of aquatic macrophytes) characteristics of wetlands and affect the distribution of fish and other species. Flow rate and eutrophication strongly impact the distribution of various biological communities because they affect the environmental conditions and the composition of food sources. Rotifers are individually small wheel animals but can form large, dominant communities among various freshwater biological communities. Therefore, they are more strongly affected by the changes in the sub-food chain, such as nutrients and phytoplankton, than their predators [15] and can be used as water quality indicators in wetlands or reservoirs [16]. Empirical studies have suggested that the density of several rotifer species, such as *Brachionus calyciflours* and *Keratella cochlearis*, increases as wetlands and reservoirs become eutrophic, whereas that of *Ascomorpha ovalis* and *Ploesoma hudsoni* decreases [16]. Changes in the community composition of primary consumers, such as rotifers, can also impact the invertebrates and fish, which are higher up in the food chain. Therefore, further research on the distribution or composition of primary consumers is required, to evaluate the environmental conditions and ecological health of wetlands.

In this research, we studied the riverine wetlands created in 2012 for restoring the natural wetlands damaged by the River Refurbishment Project in Korea. Since their creation, these riverine wetlands have been threatened by the damage and disturbance caused by the lack of follow-up investigations and the ambiguity of maintenance and management entities. Past research on these wetlands has addressed topographical changes, such as river islands and sandbars [17], the functional evaluation of the wetlands using a hydrogeomorphic approach [18], and the evaluation of biological indices [19], but it remains insufficient. The biological and riverside environments of these wetlands require urgent evaluation and continuous maintenance and management. In this study, we aim to (1) evaluate the differences in water quality according to the differences in the hydrological factors of the created riverine wetlands and (2) investigate whether the changes in water quality with variations in hydrological factors affect the composition of the rotifer community. The results of this study are expected to provide guidance for planning for the construction of new wetlands.

## 2. Materials and Methods

### 2.1. Study Sites

Freshwater ecosystems in Korea are divided into five river basins (Han, Nakdong, Geum, Yeongsan, and Seomjin basins), which directly or indirectly affect their tributaries and riverine wetlands. The Nakdong River, which includes the survey area, is in the southeastern Korean Peninsula and the second-largest river in Korea, after the Han River. This freshwater ecosystem is a temperate system and has four distinct seasons (spring, summer, autumn, and winter). The mean annual rainfall in Korea is ca. 1150 mm and mostly falls in summer, with more than 60% of the annual rainfall occurring from June to early September [20,21]. Summer-concentrated rainfall negatively affects the seasonal distribution of biological communities [22,23]. To exclude the effect of summer rainfall in this study and to understand the unique characteristics of each wetland, we conducted the survey in spring (May to June).

The area near the Nakdong River has poor drainage, and flooding for extended periods created various sizes of riverine wetlands in the area. However, most of them were lost due to human activities, such as conversions to agricultural land and residential areas and road and embankment construction [9]. In addition, the River Refurbishment Project reorganized the river section and the surrounding riverside in 2012. This project damaged or destroyed the remaining wetlands, but new wetlands were artificially created to replace them.

**Figure 1:**
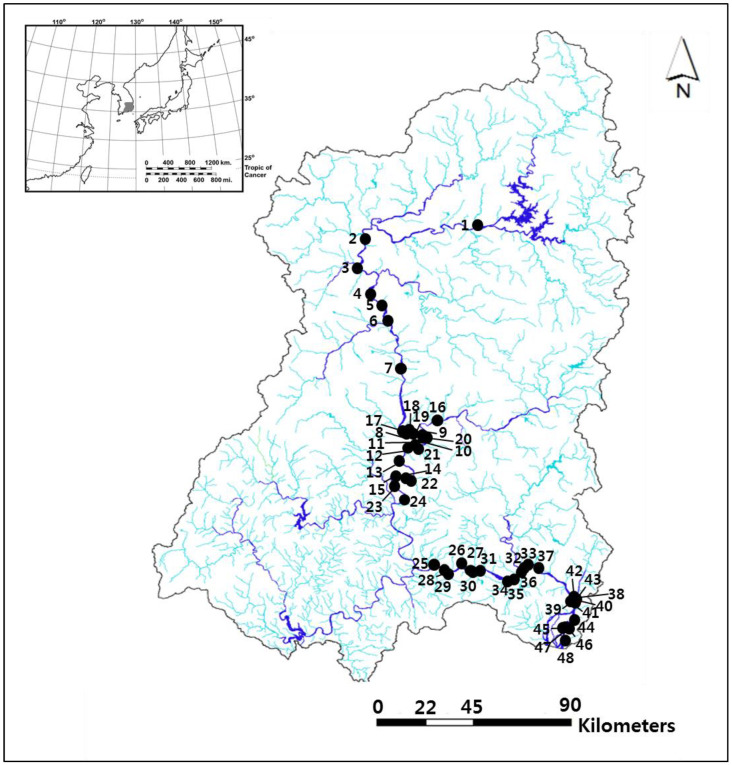
Map of the study area in the Nakdong River basin. The study sites are 48 created riverine wetlands and are represented by a closed circle (●). The upper-left inset indicates the Korean Peninsula and study area.

Table 1 summarizes some of the main morphometric and hydrological factors of the study sites. The hydrological factors were extracted from a study [24] conducted in 2017. “in/outflow function,” “shore stability,” and “water source” variables were evaluated, using three categories for each. The in/outflow function of each wetland was categorized into “good” when there were both inflow and outflow, “moderate” when there was either inflow or outflow, and “poor” when there was no inflow or outflow (wetlands with inflow and outflow ports clogged by sedimentation or plant debris were also categorized as poor). Shore stability was categorized as good if the risk of erosion or collapse was low and poor if the shore was easily eroded by water flow or showed signs of erosion. If it was neither good nor poor, it was categorized as “moderate.” Finally, the water source was categorized as “drainageway” when a drainage channel built for agricultural or commercial purposes was the main water source of the wetland, “rain/groundwater” when there was no separate water source, and “stream” if a stream or small waterway was the main water source. A total of 48 riverine wetlands were created in the Nakdong River basin (Figure 1).

### 2.2. Monitoring Strategy

We investigated the environmental variables and rotifer communities in 48 artificially created riverine wetlands in the Nakdong River basin in spring 2017 collecting three replicate samples. Eleven environmental variables (water temperature, pH, DO, conductivity, turbidity, total nitrogen (TN), total phosphorus (TP), chlorophyll *a* (Chl-*a*), depth, velocity, and macrophyte coverage) were measured at each study site. A DO meter (model 58; YSI Inc., Yellow Springs, OH, USA) was used to determine water temperature and DO, and an Orion 250A pH meter (Orion Research Inc., Boston, MA, USA) and a conductivity meter (model 152; Fisher Scientific, Hampton, NH, USA) were used to measure pH and conductivity, respectively. Velocity was determined using a hydrometer (Flowatch, JDC Electronic), and macrophyte coverage was estimated as a percentage after placing a 1 m × 1 m quadrate at the water quality measurement point.

To measure the other variables, 2 L of 0.5 m deep water was collected in a water collection bottle and transported to the laboratory. Turbidity was measured using a turbidimeter (Model DRT 100B, HF Scientific, Inc., Fort Meyers, FL, USA). To measure the Chl-*a* concentration, the water samples were filtered through 0.45 μm mixed cellulose ester membrane filters (A045A047A; Advantech Co. Ltd., Taipei, Taiwan). The filtered membranes were placed in cold 90% acetone in the dark, at 20 °C, for 4 h. To improve extraction, the cells were disintegrated for 2 min in an ultrasonic bath. To remove cell debris and filter particles, the pigment extract was centrifuged at 5000 rpm for 5–10 min. The extinction coefficient was estimated at 600 and 750 nm using a spectrophotometer (Japan Fantec Research Institute, Shizuoka, Japan), with the sample placed in a 1 cm glass cuvette [25]. The concentration of Chl-*a* was estimated using the following formula:

Chl-*a* = 11,403 × (A600 − A750) × Va × Vb^−1^ (1)

where Va is the extract volume (mL) and Vb is the sample volume (mL). We also determined TN and TP spectrophotometrically, based on the methods described by Wetzel and Likens [25].

To collect rotifer samples, water was collected in a 10 L water sampler (length: 20 cm; width: 30 cm; height: 70 cm) and filtered through a plankton net (32 µm mesh size). The filtrates were fixed in sugar formalin (final concentration: 4% formaldehyde) [26]. The rotifers were counted and identified at the species level using a Zeiss Axioskop 40 microscope (Zeiss, Göttingen, Germany) at 200× magnification and the classification key prepared by Mizuno and Takahashi [27] and Thorp and Covich [28].

### 2.3. Data Analysis

Nonmetric multidimensional scaling (NMDS) was used to examine rotifer distribution patterns according to variations in environmental variables. The NMDS ordination plots were generated based on the Euclidean distance between the study sites, and goodness of fit was assessed in terms of loss of stress. Each variation was log-transformed after being assessed for normality using the Shapiro–Wilk test. The stress value for the two-dimensional solution was 0.156, which is lower than the generally accepted maximum stress value of 0.2 [29]. The significance of the fitted vectors was assessed using 3000 permutations, with *p* < 0.05 considered significant. NMDS ordination was conducted in R package “vegan” (v.2.5-3) [30].

Multivariate analysis of variance (MANOVA) was used to identify differences in the environmental variables according to hydrological factors (i.e., in/outflow function, shore stability, water source, and wetland type). Tukey’s test was used for additional post hoc comparison analysis to verify statistically significant differences. Statistical analyses were conducted using SPSS for Windows ver. 20.0 (IBM Corp., Armonk, NY, USA, released in 2011). Differences and relationships were considered significant at *p* < 0.05.

## 3. Results

### 3.1. Environmental Variables

We observed small differences in most environmental variables among 48 wetlands (Table 2). The coefficients of variation (standard deviation/mean × 100) of most environmental variables, except water depth and flow velocity, were less than 100. The coefficient of variation of the flow velocity was 238, indicating the largest difference between the wetlands. However, with only a few exceptions, the wetlands had an almost stagnant flow, with velocities < 1 m/s.

Some environmental variables were clearly influenced by the four hydrological factors (in/outflow function, shore stability, water source, and wetland type) of each wetland (Table 3). Wetlands with poor inland/outflow function and shore stability variables showed higher TN, TP, and Chl-*a* concentrations than those with good or moderate values. The difference between the three environmental variables (TN, TP, and Chl-*a*) for inland/outflow function and shore stability factors was statistically significant (MANOVA, *p* < 0.05). In addition, the in/outflow function had a strong effect on DO and turbidity as well as TN, TP, and Chl-*a*. TN, TP, and Chl-*a* were also influenced by the water source variable. The values of these environmental variables were high in wetlands where water sources were categorized as drainageways. For the other two types of water sources (stream and rain/groundwater), the environmental variables showed relatively low values and the differences between the two source types were small. Unlike other hydrological factors, wetland types did not differ in most environmental variables. Only turbidity was higher in the pond type than in the other wetland types.

### 3.2. Rotifer Distribution

**Figure 2:**
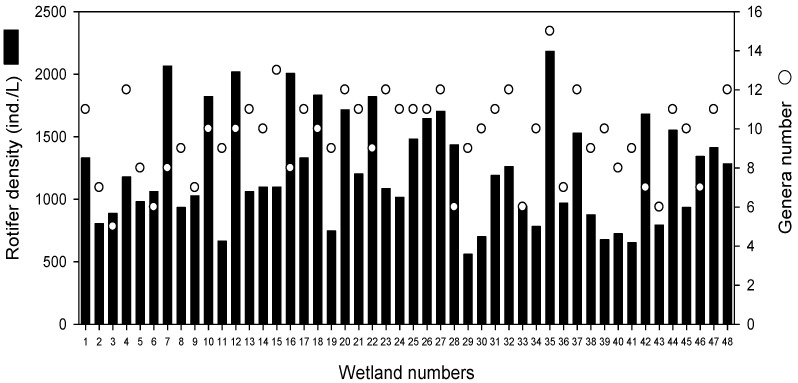
Density and genera number of rotifers in 48 artificially created riverine wetlands.

Small differences in the rotifer density and genera number were observed among 48 wetlands (Figure 2). The density of rotifers ranged from 560 to 2182 ind./L and was high (>2000 ind./L) in wetlands 35, 7, 12, and 16. In contrast, wetlands 29 and 39 were found to have the lowest density of rotifers (<700 ind./L). Wetlands 35 and 15 were supported by the highest number of rotifer genera, with more than 13 genera; most of the remaining wetlands had 10–12 genera. Wetlands 3, 6, 28, 33, and 43 were the only ones with less than seven genera.

**Figure 3:**
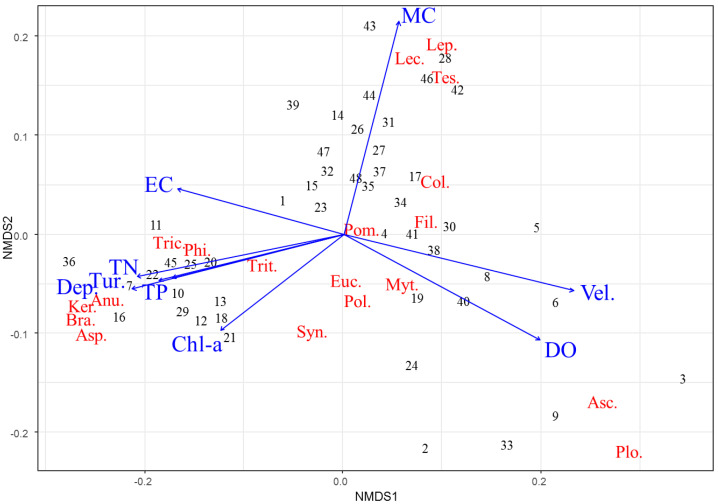
Nonmetric multidimensional scaling (NMDS) of 20 rotifer genera (red text) and 48 artificially created riverine wetlands (numbers). The blue arrows represent the associations with environmental variables: DO, dissolved oxygen; EC, conductivity; Tur., turbidity; TN, total nitrogen; TP, total phosphorus; Chl-*a*, chlorophyll *a*; Dep., depth; Vel., velocity; MC, macrophyte coverage; Anu., *Anuraeopsis*; Asc., *Ascomorpha*; Asp., *Asplanchna*; Bra., *Brachionus*; Col., *Colurella*; Euc., *Euchlanis*; Fil., *Filinia*; Ker., *Keratella*; Lec., *Lecane*; Lep., *Lepadella*; Mit., *Mytilina*; Phi., *Philodina*; Plo., *Ploesoma*; Pol., *Polyarthra*; Pom., *Pomplholyx*; Syn., *Synchaeta*; Tes., *Testudinella*; Tric., *Trichocerca*; Trit., *Trichotria*.

The NMDS analysis specified 20 dominant genera of rotifers that were affected by environmental variables (Figure 3). *Keratella*, *Brachionus*, *Asplanchna*, *Anuraeopsis*, *Trichocerca*, and *Philodina* were found in wetlands with high TN, TP, turbidity, depth, and Chl-*a*. In contrast, *Ascomorpha* and *Ploesoma* were found in wetlands with high DO and flow velocity. High densities of *Lepadella*, *Lecanec*, and *Testudinella* were found in wetlands completely covered by macrophytes. The distributions of the remaining genera (*Euchlanis*, *Polyarthra*, *Synchaeta*, *Colurella*, and *Trichotria*) were relatively irregular.

## 4. Discussion

### 4.1. Hydrological Factors and Environmental Variables

The nutrient content (TN and TP) and Chl-*a* concentrations in 48 artificially created riverine wetlands located in the Nakdong River basin were affected by three hydrological factors—in/outflow function, shore stability, and water source (Table 3). However, changes in nutrient and Chl-*a* concentrations in each wetland did not occur simultaneously. Existing studies suggest that Chl-*a* abundance increases in environments with high nutrient levels [31,32]. In this study, the relationship between the two factors could be easily determined because only a specific period in the seasonal dynamics of nutrients and Chl-*a* in each wetland was monitored, and most of the wetlands with high nutrients were also rich in Chl-*a*. The in/outflow function was a factor related to the connectivity of each wetland to the mainstream of the Nakdong River, affecting the water circulation of each wetland. Nutrients and Chl-*a* showed relatively low concentrations in wetlands with an active throughflow of mainstream water via functional in/outflow. In contrast, wetlands with no in/outflow or low function (i.e., clogging by soil and plant deposition) lacked water flow diversity and stagnated, resulting in frequent eutrophication. Empirical studies have also suggested that isolated riparian wetlands can continuously accumulate nutrients because large amounts of nutrients are introduced by runoff from the surrounding land but are not discharged [33]. However, among the constructed wetlands in the study area, those with no in/outflow function were almost absent, and most of the wetlands evaluated as poor had degraded in/outlet function. Sand, generated by the erosion of unstable shorelines, can be continuously deposited in the in/outlet, or excessive growth of aquatic macrophytes caused by a decreased flow can induce clogging of the in/outlet, isolating the wetland from the surrounding environment. Wetlands with low plant cover rates or sandy shores (poor shore stability) had high nutrient and Chl-*a* concentrations. Therefore, it is necessary to establish a plan to secure connectivity with the mainstream through continuous management of in/outflow in wetlands for which in/outflow function was evaluated as poor.

Inflow water sources were also important factors to determine the nutritional status of each wetland. Wetlands, where the major water source was a drainageway, had higher concentrations of TP and TN than those with other source types (rain/groundwater and streams). Most of the drains discharge water derived from agricultural land into the main streams around the river, and excessive fertilizer use in this area induces high nutrient inflow into the wetlands that use these drains as major water sources. Different water sources can be used to control the nutritional status of these wetlands, but it would be difficult to change the hydrological factors owing to the complexity of the physical structures involved. These wetlands have higher elevations than the mainstream and tributaries of the Nakdong River, making it physically impossible for water sources other than drainage channels to enter them. Therefore, wetlands that use drainageways as their main water sources are vulnerable to contamination or eutrophication. In addition, because water-rich drainage is only experienced at certain times, the inflow of such water sources is intermittent. Thus, these wetlands cannot maintain a constant water level and experience rapid variations in seasonal water levels.

### 4.2. Influence of Environmental Variables on Rotifer Community Composition

The influence of the hydrological factors of each wetland on the environmental variables contributed to the different distribution patterns of rotifer communities. In general, rotifer communities react sensitively to the state of their habitats, and clear differences in species or communities are often observed depending on whether the system is oligotrophic or eutrophic [16]. Therefore, existing studies have suggested that some rotifer species or genera can be used as indicators to monitor the water quality and nutritional status of lakes and wetlands [34,35]. In general, habitat quality acts as a determinant of rotifer community distribution patterns, which are mostly affected by the quality and amount of food sources such as phytoplankton and bacteria than by predation [36,37]. In this study, *Keratella*, *Brachionus*, and *Trichocerca* were mainly distributed in wetlands with high TP and TN, which is consistent with the results of the previous studies (Figure 3). *Ascomorpha* and *Pleoesoma* were mainly observed in low-nutrient wetlands and also in wetlands with better water quality (Figure 3) [38,39].

Independent of water quality variables, the abundance of rotifers such as *Lepadella*, *Monostyla*, *Lecane*, and *Testudinella* was strongly related to the coverage by aquatic macrophytes. Previous studies also reported that these four species exist in areas rich in aquatic macrophytes [5,40]. In wetlands, aquatic macrophytes complicate the physical structure of the habitats of these species, which reduces predation [41,42]. However, rotifers, which are not overly impacted by predation, tend to use aquatic macrophytes as habitats rather than shelters to avoid predators. Choi et al. [43] suggested that the leaf or stem surface of aquatic macrophytes provides an appropriate substrate for epiphytic microinvertebrates. Moreover, epiphytic plankton attached to aquatic macrophytes are appropriate food sources for rotifers [44]. Areas well covered by aquatic macrophytes are also used as shelters for protection against physical disturbances, such as rainfall. In the Upo Wetlands in Korea, the adhesion rate of *Lepadella*, *Monostyla*, and *Lecane* to the leaves and stems of aquatic macrophytes was found to increase during rainfall [45], and this result was interpreted as a defensive reaction to prevent them from being swept away by strong waves during heavy rainfall. These previous findings explain the close relationship observed between aquatic macrophytes and rotifers in this study (Figure 3).

We did not assess the characteristics of rotifer communities according to the hydrological factors of each wetland in this study because the rotifer community is more likely to be determined by the changes in environmental variables caused by hydrological factors than by the hydrological factors themselves.

Previous studies have also suggested that changes in the chemical characteristics of water bodies caused by various factors strongly influence the distribution of small invertebrates such as rotifers [46,47]. Hydrological factors such as in/outflow function, shore stability, and water source caused changes in nutrient and Chl-*a* concentrations, and these factors likely determined the species composition and distribution of the rotifer community. This sequential chain determined the flow of this study. Therefore, the species composition of rotifer communities is important to understand the environmental variables of wetlands and can be used to assess the state of the artificially created riverine wetlands. Continuously monitoring rotifer communities can generate data to help maintain the function of wetlands.

### 4.3. Wetland Management Strategy for Securing Biodiversity

Natural wetlands have undergone various hydrological changes over decades or hundreds of years, leading to the stabilization of topographic structures and biological communities. However, artificial wetlands have a relatively short formation period, and some are not constructed based on the hydrological factors (e.g., water sources or drain function), making it difficult to maintain their functions and characteristics. We propose two main conservation and management strategies, based on the results of this study: (1) improving the in/outflow structure of wetlands connecting them to secure water sources and (2) maintaining water quality, i.e., reducing turbidity by improving shoreline stability. Most artificially created riverine wetlands in the Nakdong River basin face difficulties in maintaining a stable water level. The absence or degradation of in/outflow function hinders the active throughflow of water sources, which necessitates the intermittent maintenance of the water level by groundwater or rainfall and leads to the deposition of organic matter and eutrophication via nutrient accumulation. Natural wetlands where the water level is intermittently maintained have the advantage of water quality improvement and purification by aquatic macrophytes. However, some created riverine wetland shorelines are covered by sand or gravel, making it difficult for aquatic macrophyte communities to develop. Shorelines composed of sandy soil frequently erode and collapse during heavy rainfall in summer, which changes the shape of the wetland (including meandering channel maintenance and edge complexity), causes water pollution, and increases turbidity. Therefore, measures for improving the soil quality covering the shoreline and plant activation using natural materials are urgently required. In some wetlands, soil from the shoreline flows into the wetland in large quantities, leading to shallow water depths and making it difficult to maintain wetland quantity. In addition, in wetlands where the main water source is agricultural drainage channels, the water source needs to be changed by improving the physical structure of the in/outflow ports. The inflow of drainage runoff water is a major factor in accumulating pollutants in wetlands and inducing eutrophic conditions. Most of the artificially created riverine wetlands carry strong characteristics of natural riverine wetlands, and it is, therefore, vital to expand their connections with the main streams and tributaries.

## 5. Conclusions

Based on our recommendations on the improvement plans for constructed wetlands, in this section, we discuss several points that need to be carefully reviewed when creating such artificial wetlands. First, wetlands should be built based on hydrological characteristics. The water source should continuously flow into the wetland to maintain the water level, and smooth water flow between the mainstream and the wetland should be secured by maintaining meandering and adjustment of the riverbed gradient. The second consideration is the prevention of a decrease in the water level caused by sedimentation and bank erosion and the blockage of in/outflow ports by plant deposition. The land around the wetlands should be maintained so that the minimum amount of soil flows into the wetland. This can be achieved by using highly viscous soil. Aquatic macrophytes should be used to control and secure water flow. Sustaining these items in the long term will help maintain the form and function of the wetland. Well-designed, preserved, and stabilized artificial wetlands will have no difficulty in functioning as wetlands and serving as new habitats for biological communities.

## Figures and Tables

**Table 1:** Hydrological factors of 48 artificially created riverine wetlands.

No.	Name	Latitude	Longitude	Type	Area (m^2^)	In/Outflow Function	Shore Stability	Water Source
1	Sugajigu	36°33′36.00″	128°40′21.60″	Branch	34,484.9	Moderate	Poor	Drainageway
2	Pungyang	36°30′51.90″	128°16′08.80″	Channel	3821.7	Good	Moderate	Stream
3	Gangchangnaru	36°25′11.20″	128°14′17.00″	Channel	27,905.4	Good	Good	Stream
4	Nakjeong	36°19′49.00″	128°17′15.70″	Pond	1334.9	Good	Good	Rain/Ground
5	Singok	36°17′59.20″	128°19′30.70″	Channel	9198.0	Good	Good	Stream
6	Wolgok	36°13′52.10″	128°21′10.50″	Branch	16,828.6	Good	Good	Stream
7	Ponam	36°04′18.50″	128°23′24.50″	Pond	25,249.9	Poor	Poor	Drainageway
8	Napjaru	35°50′53.00″	128°25′02.70″	Riparian	382,007.2	Good	Moderate	Stream
9	Mulsaesori	35°50′34.20″	128°26′51.60″	Channel	4987.8	Good	Good	Stream
10	Dorang	35°50′17.70″	128°27′12.60″	Pond	2668.8	Poor	Poor	Drainageway
11	Dasan	35°48′39.00″	128°27′10.20″	Channel	379,015.3	Poor	Poor	Drainageway
12	Baesuro galdae	35°48′47.00″	128°25′36.00″	Pond	30,985.1	Poor	Poor	Drainageway
13	Seongsan	35°45′56.60″	128°23′08.30″	Channel	10,612.5	Poor	Poor	Rain/Ground
14	Oksanbaesuro	35°41′59.00″	128°23′56.30″	Riparian	76,227.7	Good	Moderate	Drainageway
15	Jamsansaetgang	35°42′19.70″	128°22′28.80″	Channel	75,350.8	Moderate	Poor	Stream
16	Seojaejigu	35°52′34.02″	128°29′10.07″	Channel	313,526.8	Poor	Poor	Drainageway
17	Habin	35°51′17.50″	128°23′47.40″	Pond	78,252.1	Good	Moderate	Stream
18	Sudal sup	35°51′11.70″	128°24′24.50″	Branch	38,303.1	Moderate	Poor	Drainageway
19	Habinseubjiwon	35°51′20.50″	128°24′38.10″	Channel	70,860.4	Moderate	Good	Stream
20	Dalseongguhado	35°50′09.20″	128°28′29.90″	Channel	53,758.1	Moderate	Moderate	Drainageway
21	Galdaejeonghwa	35°48′21.70″	128°27′28.10″	Pond	67,892.7	Good	Moderate	Rain/Ground
22	Chacheon	35°41′26.20″	128°25′03.10″	Channel	123,196.3	Poor	Moderate	Drainageway
23	Hyunpungsudal	35°41′09.90″	128°21′24.30″	Channel	153,402.1	Moderate	Moderate	Stream
24	Guji	35°36′52.40″	128°23′20.20″	Channel	23,469.6	Good	Good	Stream
25	Byeoksowon	35°24′12.20″	128°30′8.30″	Pond	6206.2	Poor	Poor	Stream
26	Unjeongcheon	35°23′39.70″	128°35′21.50″	Riparian	220,687.5	Moderate	Moderate	Stream
27	Hajungdo	35°22′44.70″	128°37′32.80″	Channel	12,628.5	Moderate	Moderate	Stream
28	Cheonghwawon	35°23′06.70″	128°31′12.80″	Channel	8933.2	Moderate	Good	Stream
29	Cheongyudo	35°22′54.30″	128°32′21.00″	Channel	38,018.0	Moderate	Moderate	Stream
30	Dorae	35°22′05.70″	128°38′06.70″	Channel	26,055.2	Good	Moderate	Stream
31	Saenarae	35°22′36.30″	128°39′18.10″	Channel	27,612.3	Moderate	Moderate	Stream
32	Miryang1	35°23′18.70″	128°50′07.90″	Channel	73,857.9	Good	Moderate	Stream
33	Miryang1-1	35°23′54.40″	128°51′21.30″	Channel	6975.2	Good	Good	Rain/Ground
34	Hanlim1	35°20′22.30″	128°46′20.00″	Channel	20,170.0	Good	Moderate	Stream
35	Hanlim2	35°20′45.00″	128°47′29.10″	Channel	135,938.4	Good	Good	Stream
36	Ttanseom	35°22′29.20″	128°49′00.00″	Channel	4208.9	Poor	Poor	Stream
37	Doyo	35°22′28.30″	128°52′18.70″	Branch	2806.1	Moderate	Moderate	Stream
38	Gimhaejigu I-1	35°16′43.50″	129°00′19.50″	Channel	6113.6	Moderate	Moderate	Stream
39	Gimhaejigu I-2	35°15′48.50″	129°00′19.60″	Channel	33,039.2	Moderate	Moderate	Stream
40	Gimhaejigu II-1	35°16′17.30″	129°00′21.60″	Pond	819,682.5	Good	Good	Rain/Ground
41	Gimhaejigu II-2	35°15′48.50″	129°00′19.60″	Channel	10,313.0	Moderate	Moderate	Stream
42	Yangsanjigu	35°17′45.70″	129°00′59.30″	Channel	66,491.4	Moderate	Moderate	Stream
43	Hoesan III	35°17′16.10″	129°00′27.90″	Branch	181,424.5	Moderate	Good	Stream
44	Hwamyeong2jigu	35°12′48.20″	128°59′57.60″	Channel	4896.7	Moderate	Poor	Stream
45	Sinduk 1-1	35°11′55.90″	128°58′29.60″	Channel	24,840.7	Poor	Moderate	Drainageway
46	Sinduk 1-2	35°11′35.50″	128°57′55.60″	Branch	27,810.8	Moderate	Moderate	Stream
47	Sinduk 1-3	35°11′23.30″	128°57′46.60″	Riparian	76,143.0	Moderate	Poor	Drainageway
48	Samlak	35°09′29.30″	128°58′04.40″	Pond	15,578.8	Moderate	Good	Stream

**Table 2:** Environmental factors in 48 artificially created riverine wetlands: WT, water temperature; DO, dissolved oxygen; EC, conductivity; Tur., turbidity; TN, total nitrogen; TP, total phosphorus; Chl-*a*, chlorophyll *a*; MC, macrophyte coverage; SD, standard deviation; CV, coefficient of variation (%).

No.	WT (°C)	pH	DO (%)	EC (µs/cm)	Tur. (NTU)	TN (mg/L)	TP (mg/L)	Chl-*a* (µg/L)	Depth (m)	Velocity (m/s)	MC (%)
1	21.2	7.2	71.5	627	8.8	2.684	0.129	1.1	1.3	0.0	26
2	29.6	7.9	121.0	350	1.5	1.430	0.019	11.0	0.5	1.1	0
3	18.7	8.6	178.0	268	2.5	1.015	0.024	3.4	0.4	2.3	0
4	25.7	7.5	90.0	478	5.9	1.750	0.031	3.3	0.9	0.4	26
5	26.9	8.3	160.0	260	2.0	1.350	0.04	0.2	0.2	1.2	15
6	24.1	7.6	189.0	256	3.0	1.260	0.025	0.1	0.4	1.5	8
7	23.8	8.5	62.0	472	21.0	5.820	0.415	99.4	3.2	0.0	0
8	26.5	7.7	156.0	238	1.1	1.370	0.032	0.2	0.8	1.2	16
9	29.3	9.1	235.0	237	1.2	0.920	0.016	0.0	0.8	2.1	0
10	20.6	8.3	44.4	359	36.0	5.153	0.218	93.2	3.4	0.0	0
11	27.4	9.7	83.4	393	9.9	2.790	0.089	2.9	2.7	0.0	0
12	28.9	9.9	67.2	462	19.0	6.460	0.380	244.3	3.0	0.0	0
13	27.2	9.7	76.2	448	13.1	2.860	0.130	11.4	1.6	0.0	0
14	29.8	9.2	89.6	468	6.7	2.530	0.112	0.3	1.5	0.8	28
15	28.4	9.4	67.2	460	8.0	2.660	0.103	0.7	1.8	0.2	37
16	19.2	8.0	42.9	704	24.0	8.069	0.654	105.0	4.2	0.0	0
17	17.0	7.8	100.1	307	8.7	1.722	0.036	0.7	0.9	0.7	31
18	17.1	7.5	49.9	656	44.0	4.098	0.239	67.1	3.4	0.3	0
19	18.7	8.3	122.4	369	3.3	1.555	0.031	0.1	0.8	0.8	8
20	25.4	9.8	88.6	707	16.0	4.048	0.141	74.8	2.1	0.0	0
21	22.2	8.4	65.8	445	13.1	3.126	0.156	39.5	3.1	0.0	0
22	23.8	8.4	72.8	532	31.0	4.754	0.104	56.2	2.8	0.21	0
23	22.5	9.5	81.2	475	6.9	2.557	0.071	1.4	0.8	0.7	27
24	20.9	8.0	220.1	345	4.2	1.132	0.036	0.4	0.6	1.7	0
25	21.4	8.2	71.0	439	21.0	3.707	0.111	24.4	2.9	0.0	0
26	25.4	7.8	110.1	243	3.8	1.82	0.071	0.2	1.2	0.6	76
27	25.1	10.0	119.8	470	4.2	2.029	0.074	0.9	1.3	0.8	68
28	22.2	9.2	77.5	455	4.9	1.971	0.065	0.7	0.8	2.0	81
29	22.8	9.0	75.9	376	9.0	2.777	0.18	2.2	2.9	0.1	0
30	21.0	8.9	111.5	433	3.5	1.589	0.061	0.1	0.6	1.1	9
31	21.2	9.5	102.8	491	4.0	2.062	0.076	0.2	1.5	0.6	62
32	22.2	9.5	81.2	362	5.1	2.403	0.081	0.5	0.9	0.6	48
33	23.5	8.4	124.0	274	1.9	1.373	0.042	0.7	0.6	1.1	0
34	20.0	8.5	108.7	312	4.3	1.738	0.024	0.3	0.5	0.5	21
35	20.2	8.2	103.3	330	4.8	1.921	0.082	0.6	1.7	0.6	98
36	19.3	8.6	65.0	492	14.3	2.876	0.117	14.8	0.8	0.0	0
37	24.8	9.8	84.7	409	11.4	2.37	0.082	0.8	1.2	0.5	65
38	19.8	8.5	149.6	225	3.1	1.481	0.026	0.2	0.7	1.2	12
39	19.4	8.1	101.7	425	9.0	2.203	0.091	1.4	1.1	0.4	15
40	19.5	8.4	146.0	366	2.8	1.481	0.025	1.0	0.4	0.9	10
41	18.3	7.9	98.0	439	4.0	1.663	0.038	0.9	0.7	0.7	14
42	21.2	7.7	105.2	282	8.5	1.838	0.046	0.2	0.4	0.8	84
43	20.4	8.1	70.4	458	10.3	2.278	0.118	0.5	1.8	0.3	37
44	19.6	8.1	85.5	471	11.3	2.245	0.095	0.9	0.7	0.6	68
45	20.4	8.2	80.4	414	9.4	2.802	0.123	9.1	2.8	0.0	0
46	22.9	8.0	98.3	370	5.1	2.029	0.096	0.4	1.2	0.4	75
47	22.4	8.0	96.0	406	6.5	2.619	0.152	0.8	1.1	0.5	56
48	21.5	7.8	127.7	560	4.5	2.104	0.069	0.2	1.2	0.7	28
CV	15.1	8.7	36.9	40.8	100.1	28.3	95.7	56.9	106.3	238.0	15.1

**Table 3:** Results of factorial MANOVA comparing the effects of four hydrological factors on environmental variables: WT, water temperature; DO, dissolved oxygen; EC, conductivity; Tur., turbidity; TN, total nitrogen; TP, total phosphorus; Chl-*a*, chlorophyll *a*. Dep., depth; Vel., velocity; MC, macrophyte coverage. The mark (*) indicates a statistically significant value.

Variables	In/outflow Function			Shore Stability			Water Sources			Wetland Types		
	df.	F	*p*	df.	F	*p*	df.	F	*p*	df.	F	*p*
WT	2	1.417	0.263	2	0.539	0.590	2	2.313	0.122	3	1.327	0.290
pH	2	0.862	0.435	2	0.437	00.651	2	2.130	0.142	3	1.144	0.352
DO	2	5.499	0.011 *	2	2.446	0.109	2	2.200	0.134	3	1.408	0.266
EC	2	2.507	0.104	2	0.593	0.561	2	3.973	0.033 *	3	2.718	0.068
Tur.	2	3.925	0.034 *	2	1.741	0.198	2	3.758	0.031	3	3.405	0.041 *
TN	2	4.389	0.016 *	2	4.105	0.018 *	2	6.438	0.006 *	3	0.816	0.498
TP	2	5.244	0.012 *	2	3.905	0.033 *	2	4.266	0.027 *	3	0.093	0.963
Chl-*a*	2	3.317	0.451	2	3.984	0.020 *	2	4.175	0.029 *	3	0.915	0.449
Dep.	2	2.211	0.132	2	1.420	0.262	2	2.641	0.093	3	0.546	0.656
Vel.	2	1.681	0.208	2	0.232	0.795	2	0.013	0.987	3	0.362	0.781
MC	2	1.962	0.163	2	0.352	0.707	2	1.427	0.261	3	1.030	0.398

## Data Availability

The data presented in this study are available on request from the corresponding author. The data are not publicly available due to restrictions on the right to privacy.
